# Knowledge, Attitude and Practice of Hand Hygiene among Healthcare Workers Caring for Children with Leukaemia in the Paediatric Oncology Ward of King Saud Medical City, Saudi Arabia

**DOI:** 10.21315/mjms2023.30.4.11

**Published:** 2023-08-24

**Authors:** Mujibah Aldawsari, Kim Lam Soh, Rosna Abdul Raman, Niazlin Mohd Taib, Ahmad Aboshaiqah

**Affiliations:** 1 Department of Nursing, Faculty of Medicine and Health Sciences, Universiti Putra Malaysia, Selangor, Malaysia; 2 Department of Medical Microbiology, Faculty of Medicine and Health Sciences, Universiti Putra Malaysia, Selangor, Malaysia; 3 College of Nursing, King Saud University, Riyadh, Saudi Arabia

**Keywords:** hand hygiene, nosocomial infections, healthcare-associated infection, leukaemia children

## Abstract

**Background:**

Hands are the most common vehicle of pathogen transmission in a healthcare setting. Therefore, hand hygiene is the leading method of reducing healthcare-associated infections. This study aimed to determine the knowledge, attitude and practice (KAP) of hand hygiene and predictors for poor hand hygiene practice among healthcare workers who care for children with leukaemia in the paediatric oncology ward of King Saud Medical City (KSMC) in Saudi Arabia.

**Methods:**

One hundred and ninety medical doctors and nurses, who were registered with the Saudi Commission for Health Specialities, were selected to participate in this cross-sectional study using a simple sampling technique. Their KAP of hand hygiene was assessed using a self-structured questionnaire and the collected data was analysed using IBM^®^ SPSS^®^ version 26.0.

**Results:**

Of the 190 healthcare workers, 74.7% were nurses and 25.3% were medical doctors. Among the participants, 53.7% (102) had good knowledge of hand hygiene, 51.6% (98) had positive attitudes towards hand hygiene and 55.8% (106) practised satisfactory hand hygiene. Bachelor education level (adjusted OR = 2.736; 95% CI = 1.101, 6.799; *P* = 0.030), postgraduate education level (adjusted OR = 6.256; 95% CI = 2.171, 18.028; *P* = 0.001), poor knowledge (adjusted OR =2.575; 95% CI = 1.263, 5.246; *P* = 0.009) and negative attitude (adjusted OR = 4.702; 95% CI = 1.263, 5.246; *P* < 0.001) were the significant predictor variables of unsatisfactory hand hygiene practice among healthcare workers.

**Conclusion:**

The performance of hand hygiene among healthcare workers is still far less than optimal, particularly in settings like oncology units. Effective programmes are needed to increase their awareness of hand hygiene KAP, while strict guidelines are needed to reduce nosocomial infections.

## Introduction

Good hand hygiene has proven effective at preventing the cross-transmission of microorganisms and lowering the number of healthcare-associated infections. Hand hygiene comprises hand washing using soap and water to remove soil and transient microorganisms and hand washing using antiseptic solutions or alcohol-based hand rubs to kill microorganisms in addition to fingernail care (1). However, despite its relative simplicity and straightforwardness, only 40% of healthcare providers comply with hand hygiene (2). Therefore, efforts to identify effective and sustainable strategies to address this problem are ongoing. The World Health Organization (WHO) has proposed scientifically-based hand hygiene guidelines, recommendations and programmes. They recommend hand hygiene at five points: i) before touching a patient, ii) before performing aseptic and clean procedures, iii) after the risk of exposure to bodily fluids, iv) after connecting a patient and v) after touching a patient’s surroundings. This has helped healthcare workers improve their understanding, training, monitoring and reporting of hand hygiene (3). Hospital-acquired infections or nosocomial infections, occur when a patient is admitted for a reason other than an infection. In many countries, hospital-acquired infections are one of the most significant public health problems. The main goal of practising efficient hand washing is to reduce the rate of hospital-acquired infections among patients, especially in high-risk categories such as leukaemia. Leukaemia-related complications such as neutropenia and leukaemia treatments such as chemotherapy and its drugs increase the risk of infections when the dosage increases and when used over a prolonged period (4). According to the WHO, hospital-acquired infections are most prevalent among children with leukaemia (5). Despite the WHO’s guidelines, recommendations and programmes, hand hygiene compliance is still suboptimal among healthcare providers (6). In Saudi Arabia, most hospital staff have moderate to poor knowledge, attitude and practice (KAP) of hand hygiene (7). Despite the prevalence of healthcare-associated infections among children with leukaemia, only a handful of studies have examined the hand hygiene KAP of healthcare workers who treat this population. Therefore, this study aimed to determine the factors affecting the practice of hand hygiene among healthcare workers who care for children with leukaemia in the paediatric oncology ward of the King Saud Medical City (KSMC) in Saudi Arabia.

## Methods

### Study Design, Sampling and Participants

A cross-sectional study was conducted at the KSMC-Paediatric Hospital in Riyadh, Saudi Arabia. KSMC, also known as Shemaisi Hospital, is a prestigious public hospital and Level One Trauma Centre in Riyadh, Saudi Arabia. It was founded in 1956 and is one of the largest tertiary care centres in Saudi Arabia, with a total bed capacity of 1,500. The medical complex houses the General Hospital and the Paediatric Hospital. Paediatric Hospital includes 241 beds and 29 short-stay beds. Every year the hospital receives more than 1,271,334 patients. The inclusion criteria included medical doctors and nurses working in the paediatric oncology ward and registered with the Saudi Commission for Health Specialties while healthcare interns and non-medical staff were excluded. The sampling frame included a list of healthcare workers who cared for leukaemia patients in the paediatric oncology ward at the KSMC in Saudi Arabia between December 2021 and January 2022. The sample size was estimated using a two-proportion sampling formula. Jayakar and Reddy (8) used the proportion of positive attitudes towards hand hygiene across multiple professions as a two-proportion variable with which to calculate the sample size. A sample size of 190 was set after considering a power of 80%, a 95% confidence level and an estimated 30% incomplete data.

### Sampling Technique

With the permission of the hospital director, the sampling list consisted of every healthcare worker involved in the care of children with leukaemia at the paediatric oncology ward of KSMC-Paediatric Hospital. A simple sampling technique that utilised Microsoft Excel was used to randomly select participants from the paediatric oncology ward between December 2021 and January 2022. The sampling unit was every healthcare worker who worked at the hospital between December 2021 and January 2022. The eligibility of each healthcare worker was determined using the inclusion criteria.

### Data Collection

Upon obtaining all the necessary permissions, a self-administered questionnaire was used to collect data from eligible healthcare workers who consented to participating in this study. After the purpose of the study had been explained, each participant was given 20 min to complete the questionnaire before they were collected. The questionnaire was designed based on studies published by the WHO (5) and Nair et al. (9). It was first developed in English then double translated into Arabic and then English by two certified translators. Arabic version questionnaire was used among Arab who cannot speak English.

The Arabic version questionnaire was verified for validity and reliability, the internal consistency value was measured using Cronbach’s alpha and the result showed an acceptable level of 0.765. The reliability of English version questionnaire was ranged from 0.54 to 0.86, based on Cronbach’s alpha (10). The questionnaire had two sections: i) the socio-demographic characteristics of the participants which included their age, gender, nationality, role or profession, level of education and work experience and ii) their KAP of hand hygiene. Fourteen items were used to assess knowledge of hand hygiene, nine multiple-choice questions and five true-or-false questions. Correct answers were coded ‘1’ and wrong answers were coded ‘0’. The maximum score of this section, if all the multiple-choice answers were counted, was 32 while the minimum was zero. The knowledge of hand hygiene rated as good or poor based on the median of the total score. Thirteen Likert scale questions were used to assess attitudes towards hand hygiene. The five Likert scale choices were 1 = strongly disagree, 2 = disagree, 3 = neutral, 4 = agree and 5 = strongly agree. Questions with negative answers were coded inversely during analysis. The maximum score of this section was 65 while the minimum was 13.

The attitude was divided into positive and negative attitudes based on the median of the total score. Twelve yes-or-no questions were used to assess the practice of hand hygiene. Correct answers were coded ‘1’ and wrong answers were coded ‘0’ during analysis. The maximum score of this section was 12 while the minimum was zero. The practice of hand hygiene was categorised based on the median of the total scores into satisfactory or unsatisfactory.

### Data Analysis

The IBM^®^ Statistical Package for the Social Sciences (SPSS)^®^ version 26.0 for Microsoft Windows (Chicago, IL, USA) was used to analyse the data. Descriptive statistics such as means, median and standard deviations (SD) (continuous variables); were used to present and summarise the data while frequencies and percentages were used to present the categorical data (categorical variables). A chi-squared test was used to determine the association between category-dependent and category-independent variables. The statistical level of significance was set at *P* < 0.05. Univariate logistic regression analysis was conducted and a crude odds ratio (COR) was determined. A variable with a significant univariate test was considered as a candidate for the multivariate analysis. The significance level for the statistics was deemed acceptable at 0.05.

## Results

Table 1 summarises the sociodemographic characteristics of the participants. The participants were aged between 23 years old and 83 years old. Most of them were aged more than 35 years old (53.2%), with a median (IQR) of age was 37 (15) years old. Most of the participants were also female (85.8%). There were more Saudi participants (51.1%) than non-Saudis (48.9). Of the 190 participants, 142 (74.7%) were nurses and 48 (25.3%) were medical doctors. Most of the participants had a bachelor’s degree (54.7%, *n* = 104). This was followed by 24.2% with postgraduate education level and 21.1% with a diploma. Most of the participants (52.1%) had 5 years to 15 years of work experience.

Table 2 provides a detailed description of the knowledge of hand hygiene among the participants. Most of the participants were able to correctly identify the number of steps involved in hand hygiene as per the WHO’s recommendations while 88.9% were able to correctly identify the constituents of hand rubs. In terms of the minimum amount of time that alcohol-based hand rubs require to kill most germs, 59.5% responded correctly (20 sec) while 18.4% answered correctly regarding the duration of handwashing as per the WHOs recommendations. Of the 190 participants, 95.8% answered that soiled hands should be washed. Most of the participants (88.4%) correctly identified transient florae that hand washing removes and 86.8% correctly identified pathogens that can be transmitted by hand. Approximately 77% correctly identified the goal of hand hygiene while 68.9% correctly answered that healthcare workers with unclean hands were the main route of cross-transmissions in healthcare facilities. Regarding alcohol-based hand rubs and handwashing with soap and water, 73.2% of the participants correctly answered that hand rubs clean hands more rapidly than handwashing. Apart from that, 37.4% correctly answered that hand rubs do not cause more skin dryness than handwashing, 47.9% correctly answered that hand rubs were no more effective against germs than handwashing and 11.6% correctly answered that handwashing and hand rubs should not be used in sequence. In terms of knowledge of hand hygiene actions that prevent the transmission of germs to patients, 90% correctly answered that hand hygiene actions must be conducted before touching a patient, 84.7% correctly answered that hand hygiene actions should be conducted immediately after the risk of exposure to bodily fluids, 15.3% correctly answered that hand hygiene actions should not be conducted after exposure to the immediate surroundings of a patient to prevent the transmission of germs to patients and 91.1% correctly answered that hand hygiene actions should be conducted immediately before performing aseptic and clean procedures. In terms of hand hygiene actions that prevent the transmission of germs to healthcare workers, 92.6% correctly answered that hand hygiene actions should be conducted after touching a patient, 94.7% answered that hand hygiene actions should be conducted immediately after the risk of exposure to bodily fluids, 20% correctly answered that hand hygiene actions should not be conducted immediately before performing aseptic and clean procedures to prevent the transmission of germs to healthcare worker and 92.1% correctly answered that hand hygiene actions should be conducted after exposure to a patient’s immediate surroundings. Most of the participants correctly answered that hand hygiene actions are required before palpation of the abdomen (87.4%), 89.5% correctly answered that hand hygiene actions are required before administering an injection, 88.4% correctly answered that hand hygiene actions are required after emptying a bedpan, 49.5% correctly answered hand hygiene actions are required following contact with a patient’s bed, 51.6% correctly answered that hand hygiene actions are required following the removal of gloves, 44.7% correctly answered the same pair of gloves cannot be used when caring for different patients even if they have been washed between patients, and 91.1% correctly answered that single-use cloth towels and paper towels can be used to dry their hands in patient care areas. In terms of knowledge on jewellery, damaged skin, and artificial fingernails or long nails, 94.7%, 86.3%, and 87.9% correctly answered that they should be avoided as they increase the likelihood of colonising harmful germs on hands, respectively, while only 27.9% correctly answered that the regular use of hand creams does not increase the likelihood of colonising harmful germs on hands.

Table 3 depicts the attitudes of healthcare workers towards hand hygiene. Of the 190 participants, 68.4% strongly agreed that healthcare workers should review the respective hand hygiene guidelines of the WHO and the Centres for Disease Control and Prevention (CDC) prior to beginning clinical training while 65.3% strongly agreed that the prevention of hospital-acquired infection is a valuable part of the role of healthcare workers. Roughly 64% strongly agreed that healthcare workers must adhere to correct hand hygiene practices at all times while 38.9% strongly agreed that emergencies and other priorities make hand hygiene difficult at times. Approximately 34% and 26.8% of the participants strongly agreed that, at times, there were more important matters to attend to than hand hygiene and that wearing gloves reduces the need for hand hygiene, respectively. Around 34% strongly agreed that they feel frustrated when others neglect hand hygiene while 41.6% strongly agreed that all healthcare workers should carry a hand rub on their person. More than half of the participants (55.8%) strongly agreed that unwashed hands can transmit diseases while roughly 57% strongly agreed that hand hygiene is the most effective way of preventing and controlling the spread of most types of infectious diseases which, in turn, reduces patient mortality rates. Furthermore, 53.2%, 60%, and 60.5% strongly agreed that infection control teams have a positive influence on hand hygiene, that infection control banners are important as they remind them to practice hand hygiene, and that the current set-up made it easy for them to adhere to and practise hand hygiene, respectively.

Table 4 presents the practice of hand hygiene among healthcare workers. Of the 190 participants, 64.7% consistently adhered to correct hand hygiene protocols while 79.5% had influenced others to adhere to hand hygiene protocols. Although 93.7% washed their hands after going to the toilet, only 61.6% washed their hands after a handshake. Approximately 83% of them washed their hands before touching a patient and 90% washed their hands before performing aseptic and clean procedures. It also shows that 98.4% washed their hands after the risk of exposure to blood or bodily fluids and 93.7% washed their hands after touching a patient. However, only 73.7% washed their hands after removing gloves. Roughly 82% washed their hands after touching a patient’s surroundings and 94.7% washed their hands if their hands looked or felt dirty. Lastly, 81.6% used alcohol-based hand rubs for hand hygiene.

Hand hygiene knowledge, attitude and practice (KAP) were categorised based on the median into poor or good, negative or positive and unsatisfactory or satisfactory, respectively. Table 5 shows the total KAP of hand hygiene. Around 54% of the participants (102 out of 190) had good knowledge of hand hygiene, 51.6% had a positive attitude, and 55.8% had a satisfactory level of hand hygiene practice.

Table 6 presents the association of sociodemographic characteristics and hand hygiene knowledge and attitude with the hand hygiene practice of healthcare workers. There was a significant association between education level (χ^2^ = 8.4, *P* = 0.004) and hand hygiene practice. In addition, there was a significant association between hand hygiene knowledge (χ^2^ = 19.6, *P* < 0.001) and hand hygiene attitude (χ^2^ = 31.9, *P* < 0.001) with the hand hygiene practice of healthcare workers. However, there was no significant association between the other sociodemographic characteristics and hand hygiene practice.

Logistic regression was applied to detect the predictors of unsatisfactory hand hygiene practice among healthcare workers. As a preliminary model, all the variables were identified using univariate logistic regression one by one independently. Bachelor education level (adjusted OR = 2.736; 95% CI = 1.101, 6.799; *P* = 0.030), postgraduate education level (adjusted OR = 6.256; 95% CI =2.171, 18.028; *P* = 0.001), poor knowledge (adjusted OR = 2.575; 95% CI = 1.263, 5.246; *P* = 0.009) and negative attitude (adjusted OR = 4.702; 95% CI = 1.263, 5.246; *P* < 0.001) were the significant predictors of unsatisfactory hand hygiene practice among healthcare workers after the adjustment for age, gender, nationality, profession/role and work experience (Table 7).

## Discussion

The 190 participants in this study were relatively young (median age [IQR] = 37 [15] years old) and most of them within the age range of more than 35 years old. Aledeilah et al. (11) conducted a study in Arar City, Saudi Arabia on a similar age group (mean age ± SD = 37.2 ± 9.6 years old), where 70% were between 30 years old and 39 years old. The mean age in another study on healthcare professionals in Makkah, Saudi Arabia was 35.9 ± 7.6 years old (12). However, the mean age ± SD of healthcare providers in a Nigerian tertiary hospital who participated in a similar study was 31.3 ± 6.8 years old, with most of the participants aged 25 years old–34 years old (13). Conversely, the healthcare workers from a Ghanaian teaching hospital who participated in a study by Amissah et al. (14) were much younger, with most of them aged between 20 years old and 29 years old. However, these variations were not age-specific. Therefore, these age ranges indicate that the healthcare workers in these studies were between 20 years old and 40 years old. The differences in mean age between the studies may be because some of the participants were new recruits and had only served for a short period of time.

Existing studies have shown that healthcare workers with good knowledge of the correct hand hygiene protocols as outlined by the WHO and the CDC have good hand hygiene practices which, in turn, prevents hospital-acquired infections and reduces the transmission of germs from contaminated hands during healthcare activities (15). This present study found that 90% of the participants were able to correctly identify the six steps of hand hygiene as per the WHO guidelines. This finding was like that of another study by Modi et al. (16). The healthcare workers’ fundamental good hand hygiene knowledge in this study was 53.7%, using the median cut-off point. This study is similar to a study conducted at King Fahd Hospital in Al-Khobar, Saudi Arabia, among healthcare workers, in which 52.2% of the participants had adequate knowledge regarding hand hygiene (17). Another study in Saudi Arabia reported that around 53% of the participants (127 out of 240) had good knowledge regarding hand hygiene (11). In contrast, the findings of this result were lower than that reported in Nigeria among healthcare workers, where 82.4% of participants had a strong awareness of hand hygiene (18). In addition, a study in Malaysia reported that around 61% of healthcare workers had good hand hygiene knowledge (19). However, the 53.7% of good hand hygiene knowledge in this study was much higher than 29%, 14% and 9% reported in Saudi Arabia and India, respectively (9, 20, 21). The difference in results reported might be due to the variety of the hospital’s education curriculum or training courses. The participants of this study had different educational levels, whereas the other studies may be limited to specific educational levels.

As beliefs can affect behaviours and be used to predict it, it is essential to keep track of the perspectives of healthcare workers on hand hygiene. Furthermore, hand hygiene practices are heavily influenced by the attitudes of healthcare workers towards hand hygiene (22). Most of the participants in this present study reported positive attitudes towards reviewing the respective hand hygiene guidelines of the WHO and the CDC, the role of healthcare workers in preventing hospital-acquired infections and adherence to correct hand hygiene practices. The current study reported that the total positive attitude toward hand hygiene was 51.6%, based on the mean of the total score. This result was similar to studies in Saudi Arabia, where more than half of the participants reported a positive hand hygiene attitude (11, 21). In contrast, a study by Bakarman et al. (23) reported that 84.8% of the healthcare workers exhibited an excellent attitude which is relatively high compared to other studies, including this study. However, studies in Arabic countries reported that most healthcare workers had a poor attitude regarding hand hygiene (11, 24). These contradictory results may be explained by differences in undergraduate curricula, which may focus and emphasise more on hand hygiene importance.

As hand hygiene is the most effective way of preventing nosocomial infections, the WHO and the CDC have each recommended hand hygiene guidelines for healthcare workers (15). This study’s overall compliance rate of healthcare workers regarding hand hygiene practice was more than the median (55.8%). Similarly, a study in Saudi Arabia among healthcare workers at Hera General Hospital in Makkah, Saudi Arabia, reported that the compliance rate of healthcare workers to hand hygiene was around 51% and had a similar hand hygiene practice of healthcare workers reported in this study with a score ranging from 65% to 94% regarding adhering to correct steps of hand hygiene every time (66%), 79.5% guide others to follow hand hygiene and 94% wash their hand after touching the patient (12). In contrast, this study’s hand hygiene compliance rate was higher than in previous studies in different countries (25–28). In addition, this study’s hand hygiene compliance rate was lower than that reported by the national hand hygiene compliance rate in the United Kingdom (29). In this study, it was found that healthcare workers in the pediatric department followed good hand hygiene practices; this could be due to their frequent contact with the most vulnerable patients to hospital-acquired infections.

The present study detected no significant association between hand hygiene practice and age among healthcare workers. Other studies supported these findings that reported no significant association between hand hygiene practice and age (11, 23, 30). Furthermore, another study reported no differences between the age groups in hand hygiene practice among healthcare workers (21). However, this study contradicts the results of a study by Ahmed et al. (28) in Karachi, which found a significant association between practice hand hygiene practice and age, where the rate of hand hygiene practice adherence was higher among the age groups of 20 years old–40 years old. Regarding gender, this study reported that 57.1% of the female had satisfactory hand hygiene practice compared to 42.9% of males; however, this association was not significant (*P* = 0.4). Similarly, studies reported no significant differences in hand hygiene practice between both genders (17, 31). However, this finding contrasts with the study by Bakarman et al. (23), where female healthcare workers reported significantly better hand hygiene practices than males. Furthermore, a study by Ahmed et al. (28) in Pakistan reported significant differences in hand hygiene practice among the two genders, where male healthcare workers adhered more to hand hygiene than females. This study had no significant association between hand hygiene practice and nationality (*P* = 0.6). However, 57.7% of the Saudi healthcare workers had good hand hygiene practice compared to 53.8% of non-Saudi. These results were consistent with the results of another study carried out in Saudi Arabia, which showed no significant association between hand hygiene practice and nationality (*P* = 0.10) (32). Previous studies did not document the significant differences in hand hygiene practice among the nationality or ethnic groups. Moreover, this study showed no statistically significant association between the profession/role of the participants and the hand hygiene practice. This result is in line with a study by Aledeilah et al. (11). However, hand hygiene practice by nurses was higher than medical doctors and no statistical significance was detected. However, this finding contrasts with the study by Karaaslan et al. (33), where nurses had a statistically significant higher adherence rate to hand hygiene practice than medical doctors. The current study found that education was significantly associated with hand hygiene practice (*P* = 0.004). The participants with diplomas had a higher good hand hygiene practice (72.5%) than the other levels of education. This result supports the findings of a study by Bayleyegn et al. (34). The result found that the majority of participants (55.5%) had a first degree and there was a significant association between hand hygiene practice and education level (*P* < 0.05). On the other hand, other studies showed no significant association between hand hygiene practice and education level (23, 32, 35). The findings in this study show no significant association between hand hygiene practice and work experience. Similarly, a study conducted among healthcare workers in Ain Shams University hospitals in Egypt reported that hand hygiene practice was not significantly associated with work experience (36). On the other hand, another study showed a significant association between hand hygiene practice and work experience, where long years of experience were significantly associated with adherence to hand hygiene (37).

The current study showed a significant association between hand hygiene knowledge and the attitude of healthcare workers toward hand hygiene practice. The findings reported that the healthcare workers’ knowledge and positive attitude regarding hand hygiene were predictors of acceptable hand hygiene practice. Most healthcare workers had good hand hygiene knowledge (70.6%) and a positive hand hygiene attitude (75.5%) had satisfactory hand hygiene practice. Previous studies have evaluated the healthcare workers’ KAP on hand hygiene, investigating the association between hand hygiene knowledge and attitude with hand hygiene practice among healthcare workers and medical and nurse students. The studies reported a significant association between hand hygiene knowledge and attitude toward hand hygiene practice (12, 23, 38, 39). Although healthcare workers’ sound knowledge and positive attitude status regarding hand hygiene are commonly related to hand hygiene practice, other studies reported that healthcare workers’ sound knowledge and positive attitude regarding hand hygiene info do not automatically transform into satisfactory hand hygiene practice (38–40). This could be due to a lack of motivation, hand hygiene beliefs or the perception among healthcare workers that emergencies and other priorities can sometimes interfere with hand hygiene practice. Lankford (41) reported that healthcare workers with good hand hygiene knowledge and attitude were significantly associated with good hand hygiene practice. The study models showed that healthcare workers’ knowledge alone did not affect hand hygiene practice, although it revealed an additional effect on healthcare workers’ attitudes. Attitudes are widely considered an appropriate concept for hand hygiene practice because of their effect on health conduct and hand hygiene practice (42). From the healthcare point of view, it is essential to change healthcare workers’ attitudes that can negatively affect the beneficial hand hygiene practice because a positive attitude has a constructive role in healthcare workers’ behaviour (43, 44). Healthcare workers’ knowledge and attitudes predicted hand hygiene practice among healthcare workers. Healthcare workers’ poor knowledge of hand hygiene tactics may predict unsatisfactory hand hygiene practices (11, 13). An intervention study to improve hand hygiene practice among healthcare workers in Indonesia showed that the average knowledge regarding hand hygiene increased. Consequently, the hand hygiene compliance rate improved significantly among healthcare workers in all hospital departments (45).

There are some limitations of this study. Firstly, as the data collection instrument was a self-report questionnaire, the participants were more likely to provide socially acceptable responses when responding. Therefore, the authenticity of the responses cannot be ensured. Secondly, as this present study was conducted at only one healthcare setting, its results cannot be generalised to all other healthcare settings in Saudi Arabia as the KAP of hand hygiene among those healthcare workers may be better or worse.

## Conclusion

This present study found inadequate hand hygiene KAP among healthcare workers at the paediatric oncology ward of KSMC in Saudi Arabia; therefore, there is still a need to introduce measures that increase the KAP of healthcare workers as it may significantly improve hand hygiene compliance among the staff and reduce the cross-transmission of infections among children with leukaemia.

## Figures and Tables

**Table 1:** Distribution of sociodemographic characteristics of the participants (*N* = 190)

Variables	Frequency	%	Median (IQR)
Age (years old)			37 (15)
≤ 35	89	46.8	
> 35	101	53.2	
Gender			
Male	27	14.2	
Female	163	85.8	
Nationality			
Saudi	97	51.1	
Non-Saudi	93	48.9	
Profession/Role			
Nurse	142	74.7	
Medical doctor	48	25.3	
Education level			
Diploma	40	21.1	
Bachelor degree	104	54.7	
Postgraduate education	46	24.2	
Work experience (years)			8 (10)
Less than 5	42	22.1	
5–15	99	52.1	
More than 15	49	25.8	

Note: IQR = interquartile range

**Table 2:** Distribution of healthcare workers’ knowledge regarding hand hygiene

	Items	Frequency	%
1.	How many steps are involved in hand hygiene as per World Health Organization (WHO)? (Six steps)		
	(a) One step	1	0.5
	(b) Six steps	17	90.0
	(c) Three steps	14	14.0
	(d) Two steps	4	4.0
2.	What are the constituents of hand rub? (70% alcohol + 0.5% chlorhexidine)		
	(a) 1% alcohol	2	1.1
	(b) 70% alcohol + 0.5% chlorhexidine	169	88.9
	(c) 0.5% chlorhexidine	19	10.0
3.	What is the minimal time needed for alcohol-based hand rub to kill most germs on your hands? (20 sec)		
	(a) 20 s	113	59.5
	(b) 3 s	12	6.3
	(c) 1 min	31	16.3
	(d) 10 s	34	17.9
4.	What is the duration of hand washing as per WHO? (1 min)		
	(a) 15 min	20	10.5
	(b) 30 s	125	65.8
	(c) 3 min	10	5.3
	(d) 1 min	35	18.4
5.	If hands are soiled, you should do? (Hand washing)		
	(a) Hand rub	8	4.2
	(b) Hand washing	182	95.8
6.	Which flora is removed by hand washing? (Transient flora [transient microbiota])		
	(a) Resident flora (normal flora)	22	11.6
	(b) Transient flora (transient microbiota)	168	88.4
7.	Which of the following pathogens can be transmitted through hands? (All)		
	(a) Methicillin-resistant *Staphylococcus aureus* (MRSA)	8	4.2
	(b) Vancomycin-resistant *Enterococci* (VRE)	11	5.8
	(c) Extended spectrum beta-lactamase (ESBL) producers	6	3.2
	(d) All	165	86.8
8.	Ultimate goal of hand hygiene is to reduce? (Healthcare associated infection)		
	(a) Healthcare associated infection	147	77.4
	(b) Infection to healthcare worker only	15	7.9
	(c) Infection to patients only	28	14.7
9.	Which of the following is the main route of cross-transmission in a health-care facility? (Healthcare workers’ hands when not clean)		
	(a) Healthcare workers’ hands when not clean	131	68.9
	(b) Air circulating in the hospital	16	8.4
	(c) Patients’ exposure to colonised surfaces (i.e. beds, chairs, tables, floors)	8	4.2
	(d) Sharing non-invasive objects (i.e. stethoscopes, pressure cuffs, etc.) between patients	55	18.4
10.	Which of the following statements on alcohol-based hand rub and handwashing with soap and water are true?		
	(a) Hand rubbing is more rapid for hand cleansing than handwashing (True)	139	73.2
	(b) Hand rubbing causes skin dryness more than handwashing (False)	71	37.4
	(c) Hand rubbing is more effective against germs than handwashing (False)	91	47.9
	(d) Handwashing and hand rubbing are recommended to be performed in sequence (False)	22	11.6
11.	Which of the following hand hygiene actions prevents transmission of germs to the patient?		
	(a) Before touching a patient (True)	171	90.0
	(b) Immediately after a risk of body fluid exposure (True)	161	84.7
	(c) After exposure to the immediate surroundings of a patient (False)	29	15.3
	(d) Immediately before a clean/aseptic procedure (True)	173	91.1
12.	Which of the following hand hygiene actions prevents transmission of germs to the healthcare worker?		
	(a) After touching a patient (True)	176	92.6
	(b) Immediately after a risk of body fluid exposure (True)	180	94.7
	(c) Immediately before a clean/aseptic procedure (False)	38	20
	(d) After exposure to the immediate surroundings of a patient (True)	175	92.1
13.	Which of the following is correct about the hand hygiene (HH)?		
	(a) Hand hygiene is required before palpation of the abdomen (True)	166	87.4
	(b) Hand hygiene is required before giving an injection (True)	170	89.5
	(c) Hand hygiene is required after emptying a bedpan (True)	168	88.4
	(d) Hand hygiene is not required following contact with a patient’s bed (False)	94	49.5
	(e) Hand hygiene is not required following the removal of gloves (False)	98	51.6
	(f) The same pair of gloves can be used when caring for different patients as long as they ate washed between patients (False)	85	44.7
	(g) Single-use cloth towels and paper towels are acceptable for drying hands in patient care areas (True)	173	91.1
14.	Which of the following should be avoided, as associated with increased likelihood of colonisation of hands with harmful germs?		
	(a) Wearing Jewellery (True)	180	94.7
	(b) Damaged skin (True)	164	86.3
	(c) Artificial fingernails or long nails (True)	167	87.9
	(d) Regular use of a hand cream (False)	53	27.9

**Table 3:** Distribution of healthcare workers’ attitude regarding hand hygiene

No.	Items	Scale				

		5 = strongly agree	4	3	2	1 = strongly disagree
1.	I believe that before starting the clinical training for healthcare worker, they must review the respective WHO and CDC guidelines for hand hygiene	130 (68.4)	34 (17.9)	10 (5.3)	4 (2.1)	12 (6.3)
2.	I believe that prevention of hospital-acquired infection is a valuable part of healthcare workers’ role	124 (65.3)	46 (24.2)	14 (7.4)	1 (0.5)	5 (2.6)
3.	I believe that healthcare workers must adhere to correct hand hygiene practices at all times	121 (63.7)	48 (25.3)	14 (7.4)	1 (0.5)	6 (3.2)
4.	I believe that emergencies and other priorities make hand hygiene difficult at times	74 (38.9)	51 (26.8)	29 (15.3)	16 (8.4)	20 (10.5)
5.	I believe that sometimes there is more important things to do than hand hygiene	65 (34.2)	46 (24.2)	28 (14.7)	16 (8.4)	35 (18.4)
6.	I believe that wearing gloves reduces the need for hand hygiene	51 (26.8)	38 (20)	31 (16.3)	20 (10.5)	50 (26.3)
7.	I feel frustrated when others omit hand hygiene	65 (34.2)	45 (23.7)	34 (17.9)	14 (7.4)	32 (16.8)
8.	I believe that every healthcare worker must carry a hand rub in his/her pocket	79 (41.6)	44 (23.2)	39 (20.5)	17 (8.9)	11 (5.8)
9.	I believe that unwashed hands can transmit the diseases	106 (55.8)	40 (21.2)	31 (16.3)	7 (3.7)	6 (3.2)
10.	I believe that hand hygiene is the most effective way to prevent and control most types of infectious diseases thus reduce patients’ mortality	108 (56.8)	43 (22.6)	25 (13.2)	7 (3.7)	7 (3.7)
11.	I believe that infection control team has a positive influence on your hand hygiene	101 (53.2)	49 (25.8)	32 (16.8)	2 (1.1)	6 (3.2)
12.	I believe that it is important to have infection control banners to remind us of hand hygiene	114 (60)	40 (21.1)	26 (13.7)	5 (2.6)	5 (2.6)
13.	I believe that adhering to and hygiene practice is easy in the current set up	115 (60.5)	35 (18.4)	27 (14.2)	9 (4.7)	4 (2.1)

**Table 4:** Distribution of healthcare workers’ practice regarding hand hygiene

Items	Correct *n* (%)	Incorrect *n* (%)
1. I adhere to correct steps of hand hygiene every time	123 (64.7)	67 (35.3)
2. I guide others to follow hand hygiene	151 (79.5)	39 (20.5)
3. I wash my hand after going to the toilet	178 (93.7)	12 (6.3)
4. I wash my hand after handshaking	117 (61.6)	73 (38.4)
5. I wash my hand before touching the patient	157 (82.6)	33 (17.4)
6. I wash my hand before performing the aseptic and clean procedure	171 (90.0)	19 (10.0)
7. I wash my hand after being at risk of exposure (blood or bodily fluids)	187 (98.4)	3 (1.6)
8. I wash my hand after touching a patient	178 (93.7)	12 (6.3)
9. I wash my hand after removing gloves	140 (73.7)	50 (26.3)
10. I wash my hand after touching patient surroundings	156 (82.1)	34 (17.9)
11. I wash my hand if the hands look or feel dirty	180 (94.7)	10 (5.3)
12. I use alcohol-based hand rubs for hand hygiene	155 (81.6)	35 (18.4)

**Table 5:** The total knowledge, attitude and practice of hand hygiene among the participants

Variables	Frequency	%
Knowledge of hand hygiene Median (IQR) = 22 (6.3), min–max (11–31)		
Good	102	53.7
Poor	88	46.3
Attitude of hand hygiene Median (IQR) = 51 (11), min–max (25–65)		
Positive	98	51.6
Negative	92	48.4
Practice of hand hygiene Median (IQR) = 11 (4), min–max (3–12)		
Satisfactory	106	55.8
Unsatisfactory	84	44.2

**Table 6:** Association between sociodemographic characteristics of healthcare workers and hand hygiene practice

Variables	Hand hygiene practice		*χ* ^2^	*P*-value

	Satisfactory	Unsatisfactory		

	*n* (%)	*n* (%)		
Age (years old)				
≤ 35	44 (49.4)	45 (50.6)	2.7	0.109
< 35	62 (61.4)	39 (38.6)		
Gender				
Male	13 (48.1)	14 (51.9)	0.8	0.410
Female	93 (57.1)	70 (42.9)		
Nationality				
Saudi	56 (57.7)	41 (42.3)	0.3	0.661
Non-Saudi	50 (53.8)	43 (46.2)		
Profession/Role				
Nurse	83 (58.5)	59 (41.5)	1.6	0.2
Medical doctor	23 (47.9)	25 (52.1)		
Education level				
Diploma	29 (72.5)	11 (27.5)	8.4	0.004^*^
Bachelor degree	58 (55.8)	46 (44.2)		
Postgraduate education	19 (41.3)	27 (58.7)		
Work experience				
Less than 5	19 (45.2)	23 (54.8)	2.5	0.283
5–15	59 (59.6)	40 (40.4)		
More than 15	28 (57.1)	21 (42.9)		
Knowledge				
Good	72 (70.6)	30 (29.4)	19.6	< 0.001
Poor	34 (38.6)	54 (61.4)		
Attitude				
Positive	74 (75.5)	24 (24.5)	31.9	< 0.001
Negative	32 (34.8)	60 (65.2)		

Notes: significance ^*^P < 0.05; χ^2^ = chi-squared statistic

**Table 7:** The predictors of unsatisfactory hand hygiene practice among healthcare workers

Variables	B^a^	SE^b^	Wald	df^c^	*P*-value	OR^d^	95% CI^e^ for OR	

							Lower	Upper
Education level								
[Diploma]	1							
Bachelor degree	1.007	0.464	4.698	1	0.030*	2.736	1.101	6.799
Postgraduate education	1.834	0.540	11.530	1	0.001*	6.256	2.171	18.028
Knowledge								
[Good]	1							
Poor	0.946	0.363	6.780	1	0.009*	2.575	1.263	5.246
Attitude								
[Positive]	1							
Negative	1.548	0.363	18.146	1	< 0.001	4.702	2.306	9.584

Notes:

[ ] = reference group;

a B = coefficient for adjusted OR;

c SE = standard error;

d OR = adjusted odds ratio;

e CI = confidence interval;

* *P*-value = significant at *P* < 0.05
